# Immediate Revascularization of A Traumatic Limb Vascular Injury associated with Major Pelvic Injuries

**DOI:** 10.5704/MOJ.1511.014

**Published:** 2015-11

**Authors:** J Hanifah, M Paiman, AS Nawfar, TMS Tengku-Muzaffar, WS Wan-Azman, WI Faisham, Yahya Sahran

**Affiliations:** Department of Orthopaedic Surgery, Universiti Sains Malaysia, Kubang Kerian, Malaysia; * Plastic Reconstructive Unit, Universiti Sains Malaysia, Kubang Kerian, Malaysia

**Keywords:** Limb vascular injury, pelvic injury, revascularization, damage control orthopaedic

## Abstract

High velocity pelvic injury with limb vascular injury poses difficulties as immediate surgery for limb reperfusion is indicated. However immediate vascular intervention deviates from conventional principles of damage control following major injuries. We present two cases of this rare combination of injuries. In both cases, early limb revascularization is possible despite presented with multiple injuries and pelvic fracture.

## Introduction

The management of high velocity polytrauma patients poses a major challenge. Damage control surgery for multiple injured patients has led to increase their survival for the past few decades. Vascular injury is challenging in the context of damage control because of the conflict between time-consuming vascular reconstruction and urgent need to alleviate the procedure before a patient sustains an irreversible physiological insult. Currently, there are no clear standard guidelines and algorithms for hemorrhage control in unstable complex pelvic fracture associated with limb vascular injury.

Patients’ consent regarding publishing this case report was obtained.

## Case Report

### Case 1

A 27-year old lady motorcyclist was involved in a head-on collision with a car and sustained multiple injuries. She was initially presented in with stable hemodynamic and hemoglobin of 7.7gm /dL. She sustained right superior and inferior pubic rami fracture and bi-columnar acetabulum fracture (Fig 1-A), comminuted mid-shaft with a neck of right femur fracture, fracture distal end left radius, and right knee dislocation (Fig 1-B). The wound debridement and external fixator of the right femur and screw fixation of the neck of right femur were performed in another hospital. The pelvic and acetabulum fracture were stabilized with pelvic external fixators. During post operation, she was intubated and sedated in ICU. However, 7 hours of post operation noted distal circulation right lower limb not palpable, CT angiogram of right lower limb revealed there was a segment of filling defect of 5 cm over right popliteal artery with good distal run-off and collaterals. (Figure 1-C)

**Fig. 1a:**
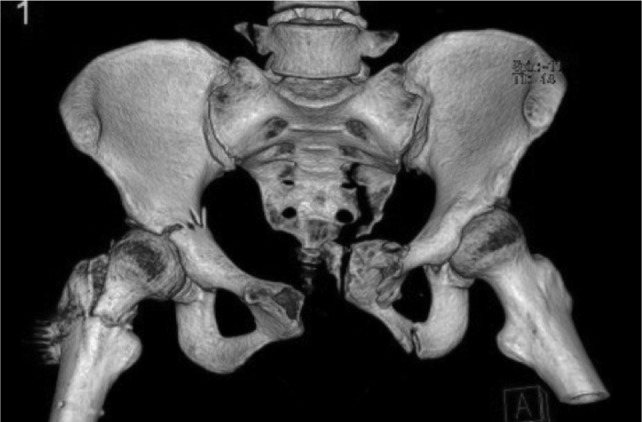
3D Computed tomography (CT imaging) revealed right bicolumnar acetabulum fracture, fracture left superior and inferior pubic rami, fracture right neck of femur.

**Fig. 1b:**
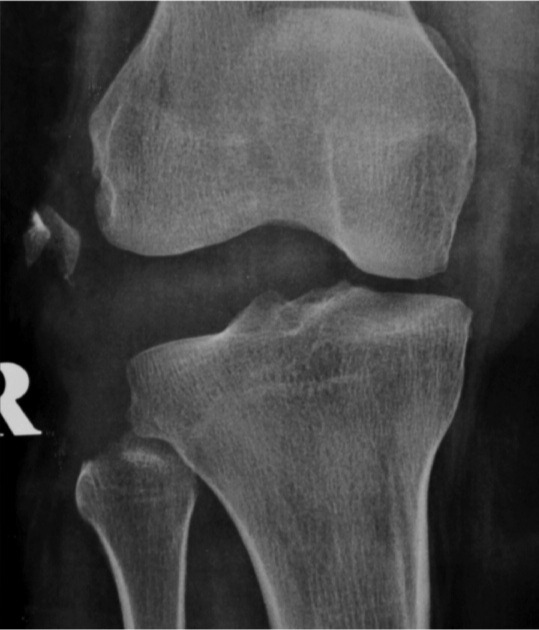
Initial radiograph right knee showing right knee dislocation.

**Fig. 1c:**
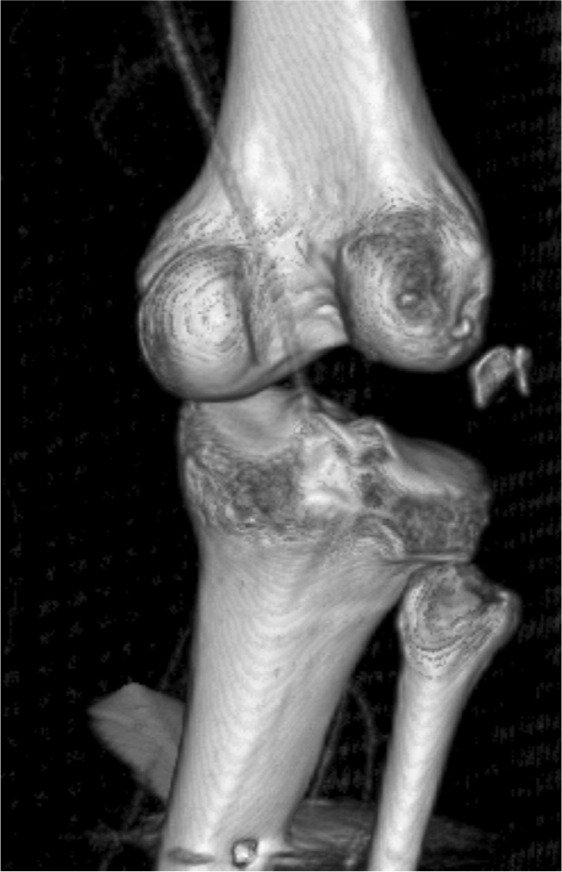
3D reconstruction Computed Tomography (CT Imaging) revealed thrombosed of right popliteal artery on dislocated knee.

Immediate stabilization of femur fractures and reverse saphenous vein graft for revascularization of the limb with prophylaxis fasciotomy were done within 15 hours. The knee dislocation was managed by posterior capsule repair and external fixation stabilization. The post-operative blood creatine phosphokinase was 7442 U/L and was increasing in trend during the first 3 days post operation, and later started decreasing in trend and normalized 7 days later. Urine output and renal function were good throughout post-operative period. She was in ICU for 12 days following vascular reconstruction, due to lung collapse and secondary infection with nosocomial infections. She required prolonged ventilation and low dose noradrenaline. Pelvic and acetabulum definitive fixation were done as a delayed procedure 23 days later through Letournel approach (Fig 1-D).

**Fig. 1d:**
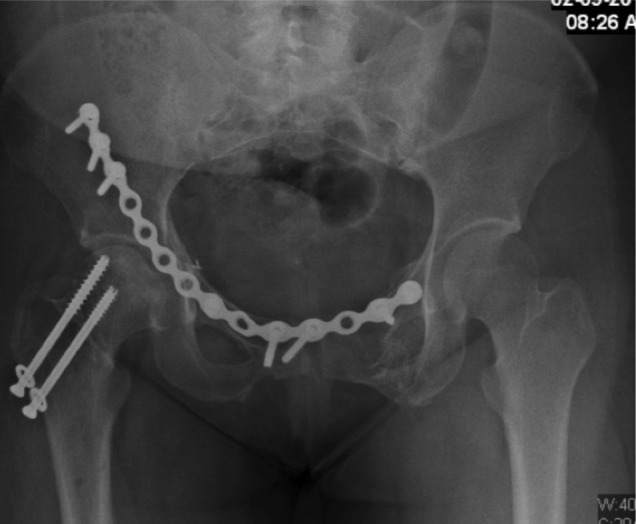
Pelvic radiograph following open reduction and ring internal fixation showing acceptable alignment, screw fixation neck of femur in situ.

After six months of post injury she could walk without crutches. All fractures healed without complication. However, she had persistent medial collateral knee instability and right foot drop secondary to peroneal nerve palsy.

### Case 2

A 23-year old young man motorcyclist was involved in a collision with a car and presented with profuse bleeding over the perineum. There was an open laceration wound over the medial aspect of left proximal thigh extending to the perianal region measuring about 10 x 2 cm with continuous blood oozing. (Fig 2-A). Computed tomography (CT) scans demonstrated severe pelvic injuries, including a left superior and inferior pubic rami fracture with left sacroiliac joint disruption (Fig 2-B). The hypovolemia was transient and was managed with fluid resuscitation and 3 pints of blood transfusion. There was proximal tibial plateau fracture in the contralateral limb with popliteal artery thrombosis of 4 cm with good collaterals and distal run-off. (Fig 2-C)

**Fig. 2a:**
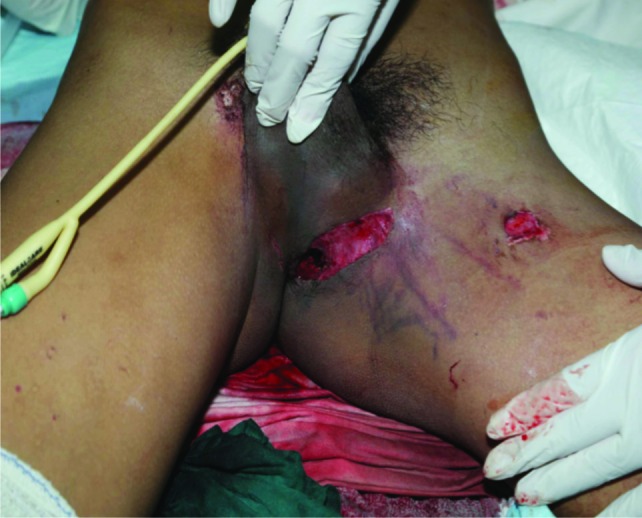
A Photograph of open pelvic fracture wound.

**Fig. 2b:**
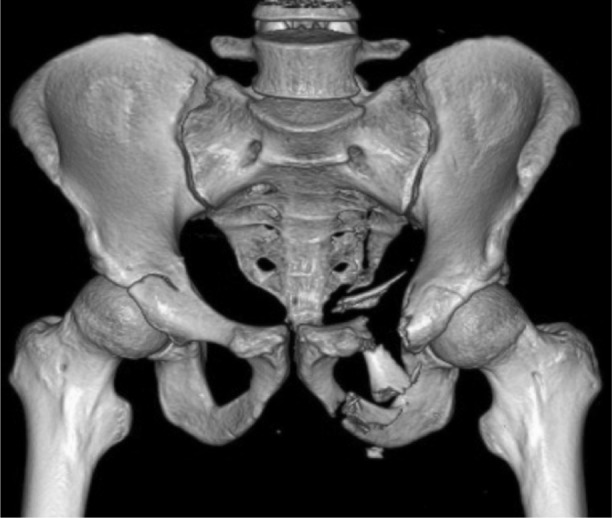
3D Computed Tomography (CT imaging) showing left superior and inferior pubic rami fracture, and opening of left sacroiliac joint.

**Fig. 2c:**
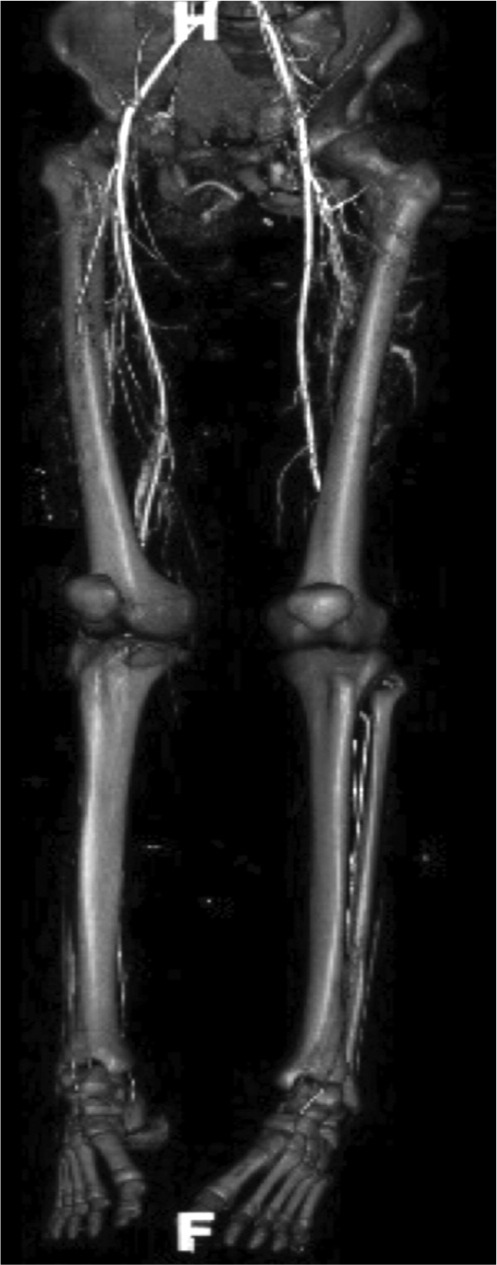
3D reconstruction Computed Tomography (CT Imaging) revealed blood collection over left periacetabular region and thrombosed of right popliteal artery with good distal run-off.

Emergency debridement of the perineal wound and pelvic packing through modified Stoppa approach were done. The bleeding was secured by clipping multiple branches of internal iliac artery and veins particularly the obturator anastomoses. The pelvis was then stabilized with a long plate. (Fig 2-D).

**Fig. 2d:**
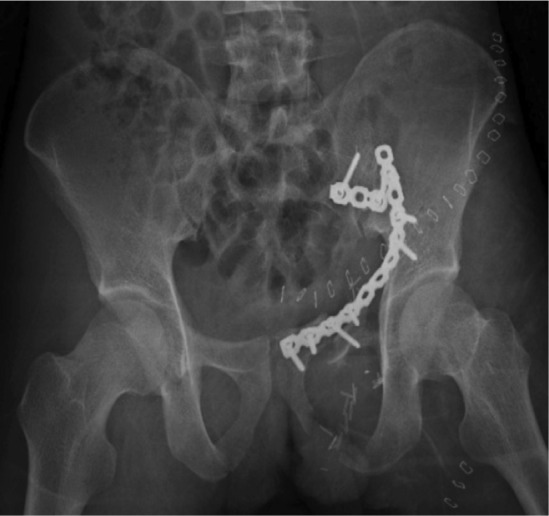
Pelvic radiograph following open reduction and ring and sacroilliac joint internal fixation.

The right popliteal vessel exploration and revascularization using saphenous vein graft were done 8 hours after injury. He was managed in ICU for 48 hours and there was no evidence of reperfusion injury. The creatine phosphokinase was 8588 U/L during first 24 hours and normalized within 3 days. No evidence of reperfusion injury and renal and lung functions were normal throughout the acute event. The total duration of hospital stay was 16 days.

## Discussion

For severely multiple injured patients who are in an ‘unstable’ or ‘in a critical’ clinical condition, damage control orthopaedic is the current treatment of choice. Its purpose is to avoid worsening the patient’s condition by the “second hit” of a major orthopaedic procedure and delay definitive repair until a time when the overall condition is optimized^1^. Both cases fall into a borderline group whereby the hypovolemia was transient and there was no major organ injury that proceeded to deterioration following early surgery. Priorities to control bleeding in open pelvic injury and vessels reconstruction to salvage the limb was the aims for both cases. Vascular injury is challenging in the context of damage control because of inherent conflict between time consuming vascular reconstruction and the urgent need to abbreviate the procedure before the patient sustains an irreversible physiological insult. The perfect vascular reconstruction also will complicate massive blood loss, coagulopathy and hypothermia that will lead to irreversible shock particularly in the present of pelvic injuries.

Hemodynamic stability and speed of surgery, early revascularization has been the gold standard for management of vascular injuries^2^. However, both cases were presented with pelvic injury that needed early damage control management. Special attention should be given to the systemic effect of reperfusion especially long ischemic time as highlighted in the first case. Prolonged and persistent myoglobulinemia and renal failure in the presence of multi-organ injuries can cause a negative impact on survival. Both patients had good collateral circulation with a short segment of thrombosis. The delayed vascular reconstruction was possible without higher risk of reperfusion injury.

Open pelvic injury may result in high-energy trauma, which places patients at risk for multiple life-threatening injuries. Mortality rate is higher in the presence of hemodynamic instability especially when continuous bleeding without tamponade occurs in open pelvic fracture. Early open packing and vascular control has been advocated to improve the survival in this difficult situation^3^. The Hannover group experiences comparing early total care and damage control orthopaedic showed that significant decrease mortality rate with early total care surgery with open packing and stabilization in complex unstable pelvic fracture^4^. However, complex pelvic injury with vascular injury associated with high mortalities despite aggressive and immediate treatment^5^.

The presence of limb vascular injury associated with complex pelvic fracture in both patients causes the dilemma, of deciding which one should be operated first. The presence of limb vascular injury can result in life-threatening injury if the bleeding is not controlled. However, once controlled, it may be given lower priority for vascular repair in order to address other life-threatening conditions. In both cases, limb vascular injuries were repaired after more than golden period of 6 hours. Previously, studies proposed by many authors, the warm ischaemic time for limb vascular injury is 6 hours to maximize limb salvage. However, some literature shows that delayed revascularization also produces acceptable limb salvage outcomes^5^.

Both cases responded to initial resuscitation with stable hemodynamic. Clinically, both cases were not in extremes that prompted the damage control orthopaedic approach to improve survival. However, in the first case limb revascularization was done early but she had complication with lung collapse and sepsis that required prolonged ventilation and ICU stay. This further delayed definitive fixation of the pelvic fractures. In the second case, the priority was to control the bleeding first because of the presence of an open wound, and loss of tamponade effect. Hemostatic control and definitive fracture stabilization of the pelvic fractures were performed first in order to minimize bleeding. Later, vascular repair and definitive fixation of limb fractures were performed when all blood parameters were stable.

In conclusion, limb revascularisation of patients with vascular injury and complex pelvic fractures is possible with acceptable morbidity. Both of these cases are very rare circumstances and will guide us for future management. Decision for surgical priority with special consideration of damage control is mandatory to achieve survival.
